# Dairy Food Intake Is Not Associated with Measures of Bone Microarchitecture in Men and Women: The Framingham Osteoporosis Study

**DOI:** 10.3390/nu13113940

**Published:** 2021-11-04

**Authors:** Courtney L. Millar, Douglas P. Kiel, Marian T. Hannan, Shivani Sahni

**Affiliations:** Marcus Institute for Aging Research, Hebrew SeniorLife, Department of Medicine, Beth Israel Deaconess Medical Center, Harvard Medical School, Boston, MA 02131, USA; courtneymillar@hsl.harvard.edu (C.L.M.); kiel@hsl.harvard.edu (D.P.K.); hannan@hsl.harvard.edu (M.T.H.)

**Keywords:** HR-pQCT, dairy food, bone, older adults

## Abstract

Previous studies reported that dairy foods are associated with higher areal bone mineral density (BMD) in older adults. However, data on bone strength and bone microarchitecture are lacking. We determined the association of dairy food intake (milk, yogurt, cheese, milk + yogurt, and milk + yogurt + cheese, servings/week) with high resolution peripheral quantitative computed tomography (HR-pQCT) measures of bone (failure load, cortical BMD, cortical thickness, trabecular BMD, and trabecular number). This cross-sectional study included participants with diet from a food frequency questionnaire (in 2005–2008 and/or 1998–2001) and measurements of cortical and trabecular BMD and microarchitecture at the distal tibia and radius (from HR-pQCT in 2012–2015). Sex-specific multivariable linear regression estimated the association of dairy food intake (energy adjusted) with each bone measure adjusting for covariates. Mean age was 64 (SD 8) years and total milk + yogurt + cheese intake was 10.0 (SD 6.6) and 10.6 (6.4) servings/week in men and women, respectively. No significant associations were observed for any of the dairy foods and bone microarchitecture measures except for cheese intake, which was inversely associated with cortical BMD at the radius (*p* = 0.001) and tibia (*p* = 0.002) in women alone. In this cohort of primarily healthy older men and women, dairy intake was not associated with bone microarchitecture. The findings related to cheese intake and bone microarchitecture in women warrant further investigation.

## 1. Introduction

Osteoporosis and low bone mass are currently estimated to be a major public health problem for almost 53.6 million US adults aged ≥50 years [1]. Dairy foods such as milk, yogurt, and cheese are good sources of bone specific nutrients, such as calcium and vitamin D, and provide more protein, calcium, magnesium, potassium, and phosphorus per kilocalorie than any other food. Yet, inconsistent findings of dairy intake and hip fracture in recent studies has led to considerable controversy surrounding potential benefits of dairy foods for bone health. Studies examining dairy intake and bone health have primarily used fracture rate [2,3,4,5,6] or areal bone mineral density (aBMD) [6,7,8,9,10], a surrogate for bone strength, which does not provide a direct measure of bone strength or bone microarchitecture [11]. A recent systematic review assigned a “B” grade or “moderate” evidence for the effect of dairy foods on bone health in middle aged and older adults [12].

Novel measurement techniques, such as high resolution peripheral quantitative computed tomography (HR-pQCT) [11] are now available to directly measure compartment-specific bone density, micro-architecture, and strength at peripheral skeletal sites of the distal radius and tibia. A few studies have highlighted the importance of dairy protein intake upon measures of bone microarchitecture in older men [8] and women [7]. However studies assessing dairy foods as a whole and their impact on novel measures of bone are sparse. In individuals from the Geneva Retiree Cohort, consuming at least 1 daily serving of fermented dairy products prevented loss of cortical BMD, area, and thickness of the radius in 433 postmenopausal women [13]. However, it is unclear if other dairy foods (such as milk) beyond fermented dairy are associated with bone microarchitecture (a key determinant of bone strength) in both men and women. Therefore, the aim of this study was to determine the association of dairy food intake with bone microarchitecture [from HR-pQCT] of the distal radius and tibia in men and women from the Framingham Offspring Study (FOS). Our hypothesis was that higher intake of dairy foods [milk, yogurt, cheese, milk + yogurt and milk + yogurt + cheese; in servings per week] would be favourably associated with bone strength and measures of bone microarchitecture in men and women.

## 2. Materials and Methods

### 2.1. Participants and Study Design

**Figure 1:**
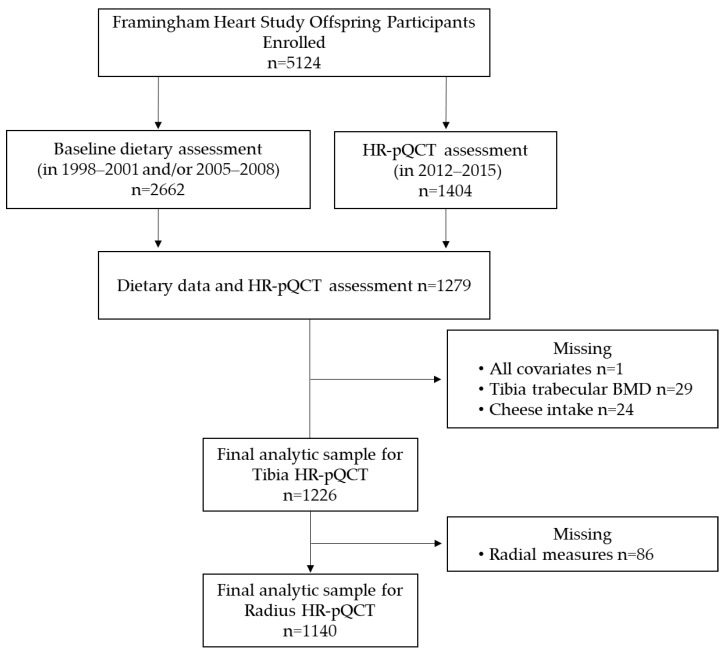
Flow chart of Framingham Heart Study Offspring cohort participants included in the HR-pQCT analysis.

This cross-sectional study included members of the FOS cohort, which are offspring of the original cohort from the Framingham Heart Study (FHS) [14]. From 1971 to 1975, 5124 offspring and their spouses of the original cohort were enrolled (age range 5–70 years at enrollment) and examined at approximately 4-year intervals to investigate risk factors for cardiovascular disease. There were 2662 participants with a valid dietary assessment (i.e., participants with non-missing or complete FFQ, based on the criteria of <12 food items left blank or energy intake >2.51 or <16.74 MJ (>600 or <4000 kcal/d) at the index examination (2005–2008) and one prior examination (1998–2001), while 1404 individuals had a valid HR-pQCT assessment in 2012–2015. Of the 1404 participants, we excluded participants with missing dietary assessment (*n* = 125), missing information on all covariates (*n* = 1), missing the tibia trabecular BMD (*n* = 29), and missing cheese intake (*n* = 24), resulting in a final analytic sample of 1226 (532 men and 694 women) for the analyses of tibia bone measures (Figure 1). Of these, 1130 participants has FFQ at both the examinations and 96 participants had an FFQ available only at the index examination (2005–2008). After excluding those missing radial measures, there was a final analytic sample of 1140 individuals for the analyses of radius bone measures (Figure 1). There were no exclusions for participants with extreme energy intake <2.51 or >16.74 MJ (<600 or >4000 kcal/d).

All FOS participants provided informed consent. This study was approved by the Institutional Review Board at the Marcus Institute for Aging Research, Hebrew SeniorLife.

### 2.2. Dietary Assessment

Usual dietary intake over the past year was assessed with a semi-quantitative Harvard food frequency questionnaire (FFQ) [15] at the index examination. Cumulative intake of dairy foods across two examinations, if available, was used as the primary exposure for all analyses given that dairy food intake did not change from one examination to another. This FFQ has been validated against multiple diet records and blood measures for many nutrients [16,17]. An earlier version of this FFQ was validated against dietary records, with food intake for seven consecutive days four times during a 1-year interval, among 173 women from Nurses’ Health Study [18]. The corrected correlation coefficients ranged from 0.57 to 0.94 for dairy products. It was also validated against a diet record and showed correlation of 0.60 (after adjustment for energy intake) [19]. Participants received the questionnaires via mail prior to their scheduled clinical visit and they were asked to bring them to the next examination, where they were reviewed by the clinical staff. The FFQ included questions on frequency with a standard serving size and 9 categories ranging from never or <1 serving/mo. to >6 servings/d. Dairy intake (servings per week) was assessed using the food list section of the FFQ. The serving size for each dairy food is as follows: skim/low or fat/whole milk (8 oz. glass), ice milk (1/2 cup), cottage or ricotta cheese (1/2 cup), other cheese (1 slice or 1 oz. serving), and yogurt (1 cup). Milk intake was calculated as the sum of intake of skim milk, low fat milk, whole milk, and ice milk. Cheese intake was calculated as the sum of intake of cottage/ricotta cheese and other cheeses (including cheeses from mixed dishes such as pizza or lasagna). Yogurt intake was estimated in servings per week. Fluid dairy intake was defined as the sum of milk and yogurt intake in servings per week and total dairy intake was defined as the sum of intake of milk, yogurt, and cheese in servings per week. Since cream intake (e.g., cream, ice cream, sour cream, cream cheese, and butter) has a high fat content and contains little to no calcium, these foods are not considered “Dairy” per the United States Department of Agriculture Food Groups [20]. Thus, we did not include cream intake in our analyses. 

### 2.3. HR-pQCT Measurements at the Radius and Tibia

Volumetric bone density and bone microarchitecture were assessed at the ultradistal tibia and ultradistal radius using HR-pQCT (XtremeCT; Scanco Medical AG, Brüttisellen, Switzerland) in 2012–2015 as described in previous studies [21,22]. The HR-pQCT device acquires CT slices with a nominal isotropic voxel size of 82 μm at the distal radius and tibia. The non-dominant forearm and right tibia were scanned, unless a participant reported having a previous fracture or had metal in the scan region of interest, in which case the contralateral side was scanned. Participants who were pregnant or unable to hold their arm and leg still for 3 min were excluded from having an HR-pQCT scan. The scan region (110 slices) began 9.5 and 22.5 mm proximal to the reference line for the radius and tibia, respectively. A quality control phantom containing rods of hydroxyapatite (HA) was scanned daily [23,24]. Images were evaluated for movement artifact using a five-point progressive movement scale. Images with the most movement (grade = 5) were excluded, whereas images rated with some movement (grade = 4), were retained for density measures [e.g., trabecular BMD (Tb.BMD), cortical BMD (Ct.BMD)] but not for architecture [e.g., trabecular thickness (Tb.Th), cortical porosity (Ct.Po)] [25]. Additionally, scans assessed by the study team as technically invalid were also excluded. Precision for HR-pQCT has been previously reported [21,26]. Linear micro–finite element analysis (FEA; Numerics88 Solutions, Inc., Calgary, AB, Canada) was performed to estimate failure load (Newtons [N]), as described [27]. Micro Finite Element Analysis (μFEA) measures integrate the contribution of cortical and trabecular morphometry, as well as bone size, and have been shown to be related to prior history of non-vertebral fracture [28,29,30]. We focused on 5 bone parameters for the HR-pQCT analysis: (1) Bone strength assessed via failure load (FL) from micro–finite element analysis, (2) Ct.BMD, (3) cortical thickness (Ct.Th) (4) Tb.BMD, and (5) trabecular number (Tb.N). The ranges of coefficients of variation (CV) are 0.7–0.9% (Ct.BMD), 0.9–1.2% (Ct.Th), 1.0–1.5% (Tb.BMD), and 3.0–3.8% (Tb.N), as previously reported [21]. 

### 2.4. Covariates

Covariates were taken from the examination closest to the time when dietary assessment was conducted and included age (years), sex, height (inches), weight (pounds), physical activity, current smoking, total energy (kcal/d), multivitamin supplement use (yes/no), calcium supplement use (yes/no), vitamin D supplement use (yes/no), and for women menopause status/estrogen use. Given that loss of estrogenic hormones in post-menopausal women is an important modulator of bone density [31], female participants were classified as 1. pre-menopausal, 2. post-menopausal and no estrogen use, or 3. post-menopausal with estrogen use. 

Height (inches) was measured while participants were without shoes and converted into meters. Weight (pounds), in light clothing was measured with a standardized balance-beam scale. Physical activity was measured with the Physical Activity Index (PAI), with scores ranging from 24–120 [32]. Total energy (kcal/d), multivitamin supplement use (yes/no), calcium supplement use (yes/no), and vitamin D supplement use (yes/no), were assessed using the FFQ’s food-list section. The smoking status of the participants was assessed via questionnaire as current cigarette smoker (smoked regularly in the past year), former smoker, or never smoker. Former and never smokers were combined into the group “noncurrent smokers”. Information on current estrogen use and menopause status (cessation of menses for at least 1 year) was obtained from self-reports on a questionnaire and verified by a medical chart review. 

### 2.5. Statistical Analysis

Baseline characteristics are presented by sex. Categorical variables are presented as percentages and continuous variables are presented as mean and standard deviation (SD). For this study we primarily conducted sex-specific analyses. However, we do present sex-combined analyses for the fully adjusted models for the entire cohort. The primary exposure variables were milk, yogurt, cheese, milk + yogurt, and milk + yogurt + cheese intake. Dairy food variables were adjusted for total energy intake by the residual method [33] and modeled as continuous variables (in servings per week). Multivariable linear regression was used to calculate regression coefficients (β) estimating the difference in bone measures associated with a 1-unit difference in dairy intake (servings per week). 

Initially, crude linear regression models were examined. Subsequent models were adjusted for age, height, weight, energy intake, current smoking, supplement use of calcium, vitamin D and multivitamins, and physical activity. Models for women were further adjusted for the for menopause status and estrogen use (pre-menopausal women, post-menopausal women with no estrogen use, or post-menopausal women with estrogen use). Given the wide age-range of the study participants, analyses were further stratified by age cut-offs (<60 years or ≥60 years). All analyses were performed using statistical software program SAS (version 9.4, SAS Institute Inc., Cary, NC, USA). To account for multiple testing, two-sided *p* values of 0.05 was divided by the number of comparisons (5 dairy foods × 2 sexes × 2 bone sites = 20). It is important to note that we did not account for multiple bone measures per bone site. Therefore, a two-sided value of 0.003 was considered statistically significant.

## 3. Results

### 3.1. Baseline Characteristics

The mean age was 64 years (SD 8; range 43–83) for men and 64 years (SD of 8; range 40–88) for women (Table 1). Ninety one percent of the men and 92% of the women did not reach the recommendation of 3 or more servings of dairy per day. Consequently, 62% of men and 43% women did not achieve recommended dietary allowance (RDA) of calcium intake for their age and gender-group. Seventeen percent of men and 63% of women used calcium supplements. More than half of men and women used multivitamin supplements, while only 8% of men and 16% of women used vitamin D supplements (Table 1). Dairy food intake was similar in men versus women with milk being the most prominent dairy food consumed. The majority of women were post-menopausal and did not use estrogen.

### 3.2. Association of Dairy Food Intake and HR-pQCT Bone Measures

In the sex-stratified, univariate models there were no associations between dairy intakes (serv/week) with any of the HR-pQCT bone measures at the radius in men or women (*p* range: radius, men: 0.062–0.982 and women: 0.021–0.978 Table 2). After adjusting for relevant confounders, higher cheese intake was associated with lower cortical BMD at the radius in women [β(SE) per 1 serv/week: −9.612 (2.739), *p* = 0.001; Table 2). Similar associations were seen at the tibia (Supplementary Table S1). There were no significant associations between dairy intake and the HR-pQCT bone microarchitecture metrics at the radius or tibia in sex-combined (Table S2). In age-stratified analyses higher cheese intake was associated with lower cortical BMD at the radius and tibia in men and women over the age of 60 years [β (SE) per 1 serv/week: −7.40 (2.59), *p* = 0.01 and per 1 serv/week: per 1 serv/week: −7.82 (2.85), *p* = 0.01, respectively; Table S3), but these associations did not reach statistical significance (*p* < 0.003). No significant associations were observed in those under the age of 60 years. 

## 4. Discussion

In this cross-sectional study, dairy food intakes were not associated with measures of bone microarchitecture at the radius or tibia in men or women, except for cheese intake. Cheese intake was negatively associated with cortical BMD at the radius and tibia in older women, but not men. No significant associations were seen with any of the bone measures from HR-pQCT in sex-combined or age-stratified analyses. 

Previous studies on this topic have largely focused on milk intake and DXA-derived aBMD [9,34,35,36,37,38,39,40,41]. A recent systematic review of 39 studies of various study designs (e.g., randomized trials, prospective cohort studies, case-control etc.) reported that there is only a moderate evidence for the effect of dairy foods on bone health in middle aged and older adults [12]. This was largely due to the mixed effects reported for the overall association between dairy intake and fractures in cohort studies [12]. In the FHS Offspring Study [38], milk was associated with hip but not spine aBMD in men and women (mean age: 55 years). However, no association was observed between dairy food intakes with either femur or spine aBMD in older men and women (aged 67–93 years) from the FHS Original cohort [9]. However, studies delineating the role of dairy food intakes with bone strength or microarchitecture have been limited in number with a focus on dairy protein intake, which was associated with failure load and stiffness in older White women [7] and with failure load, cortical thickness, and trabecular number in older men [8]. However, studies relating dairy foods with bone strength or bone microarchitecture have been limited. 

To date only one other study has reported directly on dairy food intake and bone microarchitecture utilizing data from post-menopausal women in the Geneva Retirees Cohort. A longitudinal study including 482 healthy postmenopausal women from the Geneva Retirees Cohort, reported that consumption (≥1 serving/week.) of fermented dairy products (including yogurt, fresh cheese, and kefir) at the baseline was associated with attenuated loss of radius total vBMD, cortical vBMD, area, and thickness compared to non-consumers [13]. These analyses highlight the importance of yogurt consumption on bone microarchitecture. Comparatively, our current study showed no significant associations with yogurt. It is important to note that yogurt consumption in this cohort was low ranging from 0.9–1.7 servings per week in men and women respectively, which may have resulted in null findings. Furthermore, yogurt typically consumed in European countries is a fermented food, whereas yogurt consumed in the United States is typically flavored and/or sweetened. In addition to the lack of association with yogurt, our study found no significant associations between any of the other dairy foods (i.e., milk and cheese) and measures of the bone microarchitecture at the radius or tibia in men or women except for cheese intake when utilizing HR-pQCT. The discrepancy in the yogurt findings could be explained by the differences in the study population. Overall, women in our current study seem to consume less dairy, were younger, used more calcium supplements and had higher total calcium intake than women in other studies, which could explain the lack of association in these women who may be largely calcium replete. 

Fermented milk products may have advantages for the gut microbiota [42], either due to dietary sugars that are known to stimulate intestinal calcium absorption or due to probiotic strains that likely mediate calcium absorption [42]. Although existing evidence in mice clearly demonstrates that the microbiome can influence bone mass and structure, the specific mechanisms behind these changes are not well understood [43]. Hence the effects of fermented dairy milk products on bone health are unclear. Few epidemiologic studies have examined yogurt and cheese in relation to bone health. Nevertheless, in previous studies in older adults from the Framingham Study higher yogurt intake was associated with higher aBMD [38], but cheese was not [9,38]. Interestingly, in our study cheese intake was also low (~3.5 servings per week or 0.5 serving per day), yet it was negatively associated with cortical vBMD at the radius and tibia in women alone. The reason for these negative associations are unclear given the low intakes but possible reasons include higher levels of saturated fat in processed cheeses, which has been associated with decreased dietary calcium absorption [44] that may lower bone mineral density [44,45]. Intake of hard cheeses and processed cheese products could be less advantageous to bone [46] due to high sodium content, particularly in processed cheese products and the acid-cured cheeses such as cottage cheese [47]. The sodium content of cheese may vary by region and processing practices (e.g., fermentation), which may influence the association seen between cheese intake and bone. Specifically, 0.5 serving of American cheese contains 2.5 g of saturated fat and 234 mg of sodium. Thus, while the recommended 2–3 servings of dairy taken as milk would provide ~315 mg sodium, a comparable intake of calcium from American cheese would increase the sodium intake to ~2500 mg and if consumed as cottage cheese would increase the sodium intake to ~5000 mg [48]. Future work is needed to confirm the effects of cheese intake on bone microarchitecture. 

Strengths of the current study includes use of a population-based cohort with data on specific types of dairy foods. This study utilized novel measures of bone derived from HR-pQCT, which provide information on bone microarchitecture. Lastly, this study describes sex-specific differences in peripheral microarchitecture in context of dairy foods. However, this study has potential limitations. Although we accounted for multiple comparisons due to sex, several dairy foods, and two bone sites, we did not account for multiple measures at each bone site. Additionally, the study population contained primarily men and women of European ancestry, which may limit generalizability. Misclassification of dietary intake might have occurred due to the use of a semi-quantitative FFQ, which typically results in bias towards null associations. However, use of cumulative intakes over several years measured using a validated FFQ will likely minimize misclassification. Residual confounding may have occurred despite our attempts to control for possible confounding factors. Moreover, the cross-sectional nature of this study cannot infer causality.

In conclusion, the results suggest that dairy food intake was not associated with bone microarchitecture in this cohort of primarily healthy older men and women. The negative association of cheese intake and cortical BMD in women needs to be confirmed in other studies.

## Figures and Tables

**Table 1:** Baseline characteristics of the men and women in the Framingham Offspring Study for Tibia HR-pQCT analysis.

Descriptive Variables ^1^	Men (*n* = 532)	Women (*n* = 694)
Age, years	64.2 ± 7.8	64.2 ± 7.9
BMI, kg/m^2^	28.9 ± 4.1	27.3 ± 5.3
Physical activity index	36.3 ± 6.4	35.3 ± 4.7
Menopause status and estrogen use		32 (4.6) 587 (84.6) 62 (8.9)
Pre-menopausal	-	
Post-menopausal, no estrogen use	-	
Post-menopausal, estrogen use	-	
Current smokers, *n* (%)	36 (7)	46 (7)
Calcium supplement user, *n* (%)	92 (17)	437 (63)
Multivitamin supplement user, *n* (%)	289 (54)	455 (66)
Vitamin D supplement user, *n* (%)	41 (8)	110 (16)
Dairy intake (servings/week)		
Milk	5.6 ± 5.3	5.5 ± 5.0
Yogurt	0.9 ± 1.7	1.7 ± 2.4
Cheese	3.5 ± 3.0	3.4 ± 3.2
Milk + yogurt	6.5 ± 5.7	7.1 ± 5.7
Milk + yogurt + cheese	10.0 ± 6.6	10.6 ± 6.4
Other dietary intakes		
Energy, kcal/d	2002 ± 650	1787 ± 587
Total calcium, mg/d	926 ± 389	1258 ± 504
Dietary calcium, mg/d	791 ± 325	776 ± 305
Supplemental calcium, mg/d ^2^	32 (0–181)	462 (100–750)
Total vitamin D, IU/d	438 ± 274	500 ± 265
Dietary vitamin D, IU/d	215 ± 116	204 ± 110
Supplemental vitamin D, IU/d ^2^	200 (0–400)	314 (114–400)
Radius		
Bone Strength		
Estimated failure load, (N)	3276 ± 577	1975 ± 395
Cortical bone		
Cortical vBMD (mg HA/cm^3^)	955.65 ± 53.82	956.60 ± 63.69
Cortical thickness (mm)	0.98 ± 0.20	0.80 ± 0.19
Trabecular bone		
Trabecular vBMD (mg HA/cm^3^)	188.01 ± 36.54	147.52 ± 38.64
Trabecular number (1/mm)	2.23 ± 0.27	1.91 ± 0.39
Tibia		
Bone Strength		
Estimated failure load, (N)	8046 ± 1246	5241 ± 934
Cortical bone		
Cortical vBMD (mg HA/cm^3^)	882.42 ± 67.14	835.72 ± 77.3
Cortical thickness (mm)	1.37 ± 0.27	1.07 ± 0.26
Trabecular bone		
Trabecular vBMD (mg HA/cm^3^)	196.63 ± 38.00	161.60 ± 36.94
Trabecular number (1/mm)	2.26 ± 0.34	1.91 ± 0.38

^1^ Presented as mean ± SD or *n* (%) for all such values. ^2^ Presented as median and inter quartile range for all such values.

**Table 2:** Association of dairy food intake with measures of radial HR-pQCT in men and women from the Framingham Offspring Study.

HR-pQCT Measures	Dairy Foods (Servings/Week)	Radius ^1^						Radius ^2^					
		Male (*n* = 488)			Female (*n* = 652)			Male (*n* = 488)			Female (*n* = 652)		
		Beta	SE	*p*	Beta	SE	*p*	Beta	SE	*p*	Beta	SE	*p*
Cortical vBMD, mgHA/cm^3^	Milk	−0.18	2.47	0.94	1.51	2.47	0.54	−0.66	2.32	0.78	2.66	2.17	0.22
	Yogurt	−0.53	3.09	0.86	3.73	2.79	0.18	0.22	2.89	0.93	0.18	2.50	0.94
	Cheese	−0.13	3.11	0.97	−7.12	3.08	0.02	−0.55	2.91	0.84	−9.61	2.74	<0.01 *
	Milk + Yogurt	0.35	2.45	0.89	3.54	2.73	0.19	−0.26	2.31	0.91	3.07	2.41	0.20
	Milk + Yogurt + Cheese	−0.43	0.42	0.30	−0.08	0.46	0.86	−0.42	0.39	0.28	−0.09	0.41	0.83
Cortical thickness, mm	Milk	0.01	0.01	0.20	0.01	0.01	0.39	0.01	0.01	0.20	0.01	0.01	0.13
	Yogurt	−0.02	0.01	0.22	0.00	0.01	0.62	−0.01	0.01	0.41	0.00	0.01	0.90
	Cheese	0.00	0.01	0.91	−0.01	0.01	0.57	0.00	0.01	0.79	−0.01	0.01	0.10
	Milk + Yogurt	0.01	0.01	0.60	0.01	0.01	0.23	0.01	0.01	0.55	0.01	0.01	0.19
	Milk + Yogurt + Cheese	0.00	0.00	0.30	0.00	0.00	0.98	0.00	0.00	0.37	0.00	0.00	0.90
Trabecular vBMD, mg HA/cm^3^	Milk	3.05	1.63	0.06	0.19	1.44	0.89	2.80	1.62	0.08	0.89	1.39	0.52
	Yogurt	2.65	2.05	0.20	−1.93	1.63	0.23	3.62	2.01	0.07	−1.55	1.65	0.34
	Cheese	1.65	2.06	0.42	−0.24	1.79	0.89	1.33	2.04	0.51	−1.10	1.76	0.53
	Milk + Yogurt	2.20	1.63	0.18	−0.56	1.59	0.72	2.01	1.63	0.21	−0.02	1.55	0.99
	Milk + Yogurt + Cheese	0.40	0.27	0.14	−0.24	0.27	0.37	0.39	0.27	0.16	−0.24	0.26	0.37
Trabecular number,1/mm	Milk	0.01	0.01	0.23	−0.01	0.02	0.74	0.01	0.01	0.31	0.01	0.01	0.70
	Yogurt	0.00	0.02	0.94	−0.03	0.02	0.07	0.00	0.02	0.89	−0.03	0.02	0.10
	Cheese	0.00	0.02	0.79	0.01	0.02	0.51	−0.01	0.02	0.60	0.00	0.02	0.91
	Milk + Yogurt	0.01	0.01	0.43	−0.01	0.02	0.44	0.01	0.01	0.51	−0.01	0.02	0.74
	Milk + Yogurt + Cheese	0.00	0.00	0.67	0.00	0.00	0.34	0.00	0.00	0.76	0.00	0.00	0.44
Estimated failure load, N	Milk	31.11	26.55	0.24	16.61	15.18	0.27	18.73	25.07	0.46	24.26	13.27	0.07
	Yogurt	25.93	35.41	0.46	−27.45	16.94	0.10	24.16	33.53	0.47	−25.66	15.42	0.10
	Cheese	58.11	33.23	0.08	7.74	19.13	0.69	47.12	31.33	0.13	−20.38	17.00	0.23
	Milk + Yogurt	25.13	26.39	0.34	7.82	16.91	0.64	13.39	25.00	0.59	8.62	14.86	0.56
	Milk + Yogurt + Cheese	0.10	4.44	0.98	−1.67	2.81	0.55	−1.23	4.18	0.77	−3.02	2.49	0.22

^1^ Crude models ^2^ Models adjusted for age, height, weight, current smoking, energy intake, calcium supplement use, vitamin D supplement use, menopause status/estrogen use (in women alone), physical activity, and multivitamin use. * *p*-value < 0.003 was considered statistically significant.

## Data Availability

Data described in the manuscript, code book, and analytic code will be made available upon request pending application to and approval by the Framingham Heart Study.

## Supplementary Materials

The following are available online at https://www.mdpi.com/article/10.3390/nu13113940/s1, Table S1: Association of dairy food intake with measures of tibial HR-pQCT in men and women from the Framingham Offspring Study, Table S2: Association of dairy food intake (serv/week) with measures of HR-pQCT in the combined sample of men and women from the Framingham Offspring Study, Table S3: Association of dairy food intake with measures of HR-pQCT stratified by age in participants from the Framingham Offspring Study.
