# Supplementary material for: Dairy Food Intake Is Not Associated with Measures of Bone Microarchitecture in Men and Women: The Framingham Osteoporosis Study
Source: Nutrients. 2021 Nov 4;13(11):3940. doi: 10.3390/nu13113940 (PMC8622947; doi:10.3390/nu13113940)
Supplement: Supplementary file 1 [file nutrients-13-03940-s001.zip › nutrients-1405857-Supplementary.pdf]

nutrients

Supplemenatary Tables

Table S1. Association of dairy food intake with measures of tibial HR-pQCT in men and women from the Framingham Offspring Study.

		Tibia [1]						Tibia [2]					
**HR-pQCT Measures**	**Dairy Foods (Servings/wk)**	**Male (n=532)**			**Female (n=694)**			**Male (n=532)**			**Female (n=694)**		
		**beta**	**SE**	**P**	**beta**	**SE**	**P**	**beta**	**SE**	**P**	**beta**	**SE**	**P**
Cortical vBMD, mgHA/cm³	Milk	-0.85	2.96	0.77	0.07	2.79	0.98	-1.66	2.68	0.53	2.52	2.44	0.30
	Yogurt	-2.14	3.44	0.53	0.04	3.20	0.99	-1.51	3.11	0.63	-2.56	2.84	0.37
	Cheese	-2.40	3.61	0.51	-4.35	3.51	0.22	-2.14	3.26	0.51	-9.42	3.10	<0.01*
	Milk+Yogurt	-0.61	2.97	0.84	-0.04	3.05	0.99	-1.83	2.69	0.50	0.57	2.67	0.83
	Milk+Yogurt+Cheese	-0.58	0.48	0.22	-0.42	0.51	0.42	-0.65	0.43	0.13	-0.43	0.45	0.34
Cortical thickness, mm	Milk	0.01	0.01	0.62	0.00	0.01	0.99	0.01	0.01	0.59	0.01	0.01	0.29
	Yogurt	-0.02	0.02	0.13	0.01	0.01	0.50	-0.02	0.01	0.27	0.00	0.01	0.85
	Cheese	0.00	0.01	0.92	0.00	0.01	0.87	0.00	0.01	0.84	-0.01	0.01	0.17
	Milk+Yogurt	0.00	0.01	0.87	0.00	0.01	0.82	0.00	0.01	0.87	0.01	0.01	0.52
	Milk+Yogurt+Cheese	0.00	0.00	0.91	0.00	0.00	0.40	0.00	0.00	0.85	0.00	0.00	0.31
Trabecular vBMD, mg HA/cm³	Milk	2.57	1.58	0.10	0.28	1.32	0.83	2.31	1.54	0.13	0.74	1.26	0.56
	Yogurt	4.16	2.05	0.04	-2.83	1.52	0.06	4.86	2.00	0.02	-1.43	1.52	0.35
	Cheese	1.79	1.96	0.36	-1.16	1.64	0.48	1.21	1.90	0.53	-2.27	1.60	0.16
	Milk+Yogurt	2.19	1.58	0.17	-0.47	1.45	0.74	2.21	1.55	0.15	0.04	1.40	0.98
	Milk+Yogurt+Cheese	0.34	0.26	0.19	-0.25	0.24	0.30	0.38	0.25	0.13	-0.31	0.23	0.18
	Milk	0.00	0.02	0.93	0.01	0.01	0.60	0.00	0.01	0.79	0.01	0.01	0.26
	Yogurt	0.02	0.02	0.21	-0.03	0.02	0.04	0.03	0.02	0.10	-0.01	0.02	0.51

Trabecular number,1/mm	Cheese	0.03	0.02	0.10	0.01	0.02	0.59	0.02	0.02	0.33	-0.01	0.02	0.50
	Milk+Yogurt	0.00	0.02	0.88	0.01	0.02	0.67	0.00	0.01	0.98	0.02	0.01	0.28
	Milk+Yogurt+Cheese	0.00	0.00	0.73	0.00	0.00	0.57	0.00	0.00	0.88	0.00	0.00	0.35
Estimated failure load, N	Milk	29.52	55.10	0.59	28.97	33.76	0.39	12.41	49.87	0.80	52.81	28.34	0.06
	Yogurt	24.97	70.59	0.72	-12.33	38.79	0.75	40.03	63.70	0.53	16.76	33.62	0.62
	Cheese	118.94	66.58	0.07	26.64	42.22	0.53	81.54	60.46	0.80	-55.26	36.10	0.13
	Milk+Yogurt	22.39	54.77	0.68	25.54	36.95	0.49	15.89	49.72	0.75	34.97	31.20	0.26
	Milk+Yogurt+Cheese	-0.85	2.96	0.77	0.07	2.79	0.98	3.40	8.05	0.67	-5.21	5.24	0.32

[1] Crude models

[2] Models adjusted for age, height, weight, current smoking, energy intake, calcium supplement use, vitamin D supplement use, menopause status/estrogen use (in women alone), physical activity, and multivitamin use.

* *P*-value < 0.003 was considered statistically significant.

Table S2. Association of dairy food intake (serv/wk) with measures of HR-pQCT in the combined sample of men and women from the Framingham Offspring Study.

HR-pQCT Measures	Dairy Foods (servings/wk)	Radius (n=1140)			Tibia (n=1126)		
		beta	SE	P-value*	beta	SE	P-value*
Cortical vBMD, mgHA/cm³	Milk	1.19	1.61	0.46	0.58	1.83	0.75
	Yogurt	0.53	1.91	0.78	-2.37	2.13	0.27
	Cheese	-5.37	2.02	0.01	-5.82	2.28	0.01
	Milk+Yogurt	1.45	1.71	0.40	-0.83	1.93	0.67
	Milk+Yogurt+Cheese	-0.26	0.29	0.36	-0.56	0.32	0.08
Cortical thickness, mm	Milk	0.01	0.01	0.06	0.01	0.01	0.29
	Yogurt	0.00	0.01	0.53	-0.01	0.01	0.48
	Cheese	-0.01	0.01	0.22	-0.01	0.01	0.49
	Milk+Yogurt	0.01	0.01	0.21	0.00	0.01	0.64
	Milk+Yogurt+Cheese	0.00	0.00	0.45	0.00	0.00	0.53
Trabecular vBMD, mg HA/cm³	Milk	1.60	1.06	0.13	1.41	0.98	0.15
	Yogurt	0.12	1.26	0.93	0.63	1.20	0.60
	Cheese	-0.07	1.33	0.96	-0.64	1.23	0.60
	Milk+Yogurt	0.90	1.13	0.42	1.05	1.04	0.31
	Milk+Yogurt+Cheese	0.06	0.19	0.74	0.02	0.17	0.89
Trabecular num-ber,1/mm	Milk	0.01	0.01	0.59	0.01	0.01	0.50
	Yogurt	-0.02	0.01	0.18	0.00	0.01	0.87
	Cheese	0.00	0.01	0.87	0.00	0.01	0.88
	Milk+Yogurt	0.00	0.01	0.93	0.01	0.01	0.46
	Milk+Yogurt+Cheese	0.00	0.00	0.52	0.00	0.00	0.39
Estimated failure load, N	Milk	22.73	13.10	0.08	35.78	26.66	0.18
	Yogurt	-9.59	15.31	0.53	19.43	31.33	0.54

Cheese	10.40	16.56	0.53	8.14	33.13	0.81
Milk+Yogurt	10.73	13.92	0.44	23.53	28.06	0.40
Milk+Yogurt+Cheese	-2.14	2.32	0.36	-1.53	4.62	0.74

Models adjusted for age, sex, height, weight, current smoking, energy intake, calcium supplement use, vitamin D supplement use, physical activity, and multi-vitamin use.

* *P*-value < 0.003 was considered statistically significant.

Table S3. Association of dairy food intake with measures of HR-pQCT stratified by age in participants from the Framingham Offspring Study.

HR-pQCT Measures	Dairy Foods (servings/wk)	Radius						Tibia					
		Age≤60 years (n=387)			Age>60 years (n=753)			Age≤60 years (n=395)			Age>60 years (n=831)		
		beta	SE	P-value	beta	SE	P-value	beta	SE	P-value	beta	SE	P-value
Cortical vBMD, mgHA/cm³	Milk	0.32	2.46	0.90	1.44	2.11	0.50	0.27	2.91	0.93	0.48	2.33	0.84
	Yogurt	-2.05	2.96	0.49	2.02	2.48	0.42	-4.38	3.34	0.19	-1.57	2.72	0.57
	Cheese	-1.38	3.19	0.67	-7.40	2.59	0.01	-2.03	3.68	0.58	-7.82	2.85	0.01
	Milk+Yogurt	-0.85	2.76	0.76	2.35	2.17	0.28	-2.27	3.21	0.48	-0.39	2.40	0.87
	Milk+Yogurt+Cheese	-0.30	0.45	0.51	-0.28	0.36	0.45	-0.36	0.53	0.50	-0.70	0.39	0.07
Cortical thickness, mm	Milk	0.01	0.01	0.12	0.01	0.01	0.20	0.01	0.01	0.40	0.01	0.01	0.31
	Yogurt	-0.01	0.01	0.47	0.00	0.01	0.83	0.00	0.01	0.85	-0.01	0.01	0.41
	Cheese	0.00	0.01	0.83	-0.01	0.01	0.09	0.01	0.01	0.59	-0.01	0.01	0.22
	Milk+Yogurt	0.01	0.01	0.57	0.01	0.01	0.21	0.00	0.01	0.77	0.01	0.01	0.51
	Milk+Yogurt+Cheese	0.00	0.00	0.55	0.00	0.00	0.71	0.00	0.00	0.32	0.00	0.00	0.96
Trabecular vBMD, mg HA/cm3	Milk	2.07	1.78	0.25	1.05	1.32	0.43	1.59	1.76	0.36	1.14	1.18	0.34
	Yogurt	-2.58	2.07	0.21	1.38	1.59	0.39	-2.47	2.05	0.23	1.91	1.47	0.20
	Cheese	-0.42	2.28	0.86	0.49	1.63	0.76	-0.84	2.20	0.70	-0.17	1.46	0.91
	Milk+Yogurt	0.01	1.95	0.99	0.90	1.38	0.51	1.07	1.92	0.58	0.82	1.23	0.51
	Milk+Yogurt+Cheese	-0.16	0.33	0.62	0.10	0.23	0.66	-0.07	0.32	0.82	0.03	0.20	0.88
Trabecular number,1/mm	Milk	0.00	0.02	0.92	0.01	0.01	0.52	0.00	0.02	0.87	0.01	0.01	0.49
	Yogurt	-0.04	0.02	0.02	0.00	0.02	0.87	-0.01	0.02	0.44	0.00	0.01	0.51
	Cheese	-0.02	0.02	0.21	0.01	0.02	0.49	0.01	0.02	0.76	0.00	0.01	0.94
	Milk+Yogurt	-0.03	0.02	0.08	0.01	0.01	0.43	0.00	0.02	0.85	0.00	0.01	0.42
	Milk+Yogurt+Cheese	-0.01	0.00	0.07	0.00	0.00	0.77	0.00	0.00	0.42	0.00	0.00	0.49
Estimated failure load, N	Milk	8.25	21.65	0.70	23.68	16.34	0.15	17.11	44.30	0.70	39.90	33.27	0.23
	Yogurt	-14.16	25.38	0.58	-8.89	19.02	0.64	-0.63	50.26	0.99	29.41	39.62	0.46
	Cheese	9.69	27.75	0.73	17.07	20.30	0.40	29.43	55.93	0.60	6.63	40.64	0.87

	Milk+Yogurt	-13.13	24.04	0.59	13.55	16.93	0.42	-6.82	48.66	0.89	31.03	34.12	0.36
	Milk+Yogurt+Cheese	-7.38	3.93	0.06	-0.75	2.84	0.79	-8.20	8.04	0.31	0.38	5.58	0.95

Models adjusted for age, sex, height, weight, current smoking, energy intake, calcium supplement use, vitamin D supplement use, physical activity, and multivitamin use.

* *P*-value < 0.003 was considered statistically significant
